# American Medical Association

**Published:** 1857-06

**Authors:** 


### AMERICAN MEDICAL ASSOCIATION.

From the fact that the number of delegates in attendance at the late meeting of the American Medical Association was nothing like so large as usual, and from the failure upon the part of the more prominent members of the profession, (with a few exceptions) to attend, it would seem that there is a waning interest in its deliberations.

The truth is, the practical working of this body clearly convinces us that elements have already been introduced into its operations, which will finally scatter it to the winds, unless more wisdom and less ambition is found in its future proceedings. As a social "re-union," and as an occasion for the presentation of contributions to science, and as an organized arrangement for the diffusion of these treasures, it has our *hearty good will*; but as an instrument for the elevation of a favored few, and as affording an opportunity for an annual volley of abuse of the medical Colleges, from disappointed aspirants for Professors' chairs, it shall ever have our *hearty condemnation.* We must confess ourselves really sick of this eternal cry about the inferiority of the medical profession of the United States. Our doctrine (and we have no hesitation in stating it) is, that the medical men of this country are fully as good as the people *deserve*, or are willing *to pay for*, and with fully as large a share of practical skill in the treatment of disease, (belonging to their country) as can be found upon the globe.

In saying this much, we do not mean to be understood as contending for the position, that the system of American Medical education is the best in the world ; but we do contend, that in the present condition of this country, you cannot support a system of a much more expensive and extended character than the present, for while it is true that many graduate and commence practice who are but indifferently prepared, they generally settle in communities who would not or could not remunerate a medical man, who had spent double or treble the time in fitting himself for his profession.

Some, more fastidious than wise, may object to the *money* view of the subject, but in this country, with the innumerable avenues to wealth, you will not find men devoting much time or money to a profession—attended at best with so many sacrifices—and affording no adequate return, in its revenue, for what has been expended upon it—this *we know* to be the fact, the prating of would-be, and over-wise *reformers*, to the contrary notwithstanding, which especially comes with a bad grace from those who are in a position to "offer medical education, without money and without price."

